# Erectile Dysfunction in Men with Chronic Obstructive Pulmonary Disease

**DOI:** 10.3390/jcm10122730

**Published:** 2021-06-21

**Authors:** Lorenzo Marinelli, Fabio Lanfranco, Giovanna Motta, Marco Zavattaro

**Affiliations:** 1 Division of Endocrinology, Diabetology and Metabolism, Department of Medical Sciences, University of Turin, 10126 Turin, Italy; lorenzo.marinelli@unito.it (L.M.); giovanna.motta@unito.it (G.M.); marco.zavattaro@unito.it (M.Z.); 2 Division of Endocrinology, Andrology and Metabolism, Humanitas Gradenigo, Department of Medical Sciences, University of Turin, 10153 Turin, Italy

**Keywords:** erectile dysfunction, chronic obstructive pulmonary disease, sexuality, hypogonadism, cardiovascular disease, mood disorders, treatment

## Abstract

Erectile dysfunction (ED) seems to be a widespread sexual issue in men affected by chronic obstructive pulmonary disease (COPD). Multiple causes appear to be involved such as hormonal imbalance, smoking habit, chronic inflammation, endothelial dysfunction, chronic hypoxia, psychiatric disorders (depression and anxiety), and medications. ED can have a significant impact on COPD men and consequently on their quality of life, which is usually already compromised. Given this situation, however, pneumologists usually do not properly care for the sexuality of COPD patients especially because men can be reluctant to talk about their intimate issues. The aim of this narrative review is to briefly summarize the evidence emerging from literature and to provide a wide point of view about sexual dysfunction in COPD men.

## 1. Introduction

Chronic obstructive pulmonary disease (COPD) is a common, preventable, and treatable disorder. It is characterized by persistent respiratory symptoms and airflow limitation due to airway and/or alveolar abnormalities [1]. Alarmingly, COPD is now the third leading cause of death globally [2]. This disease occurs as a result of long-term exposure to harmful particles or gases (mainly cigarette smoke) that leads to an increased inflammatory response in the airways [1]. These noxious factors cause chronic lung inflammation that progressively extends to a systemic involvement. The natural history of the disease is characterized by progressive worsening of respiratory function. Sometimes disease exacerbations occur, with acute dyspnea and respiratory distress that can accelerate disease progression and result in hospital admission and death [3].. Furthermore, considering COPD as a systemic disease, it is accompanied by impactful comorbidities, such as cardiovascular diseases, cachexia, skeletal muscle dysfunction and loss, osteoporosis, anemia, obstructive sleep apnea, lung cancer, diabetes mellitus, metabolic syndrome, depression, and anxiety [1,4,5]. This overall situation negatively affects the quality of life (QoL) of patients with COPD [6,7].

Erectile dysfunction (ED) is defined as the inability to achieve or maintain an erection that is sufficient for satisfactory sexual performance [8]. It is a common medical disorder that primarily affects men older than 40 years and it progressively worsens with age [8]. In more than 80% of cases, ED is related to an organic etiology. Findings from several cross-sectional and longitudinal studies have linked the development of ED to an unhealthy lifestyle (smoking, obesity, and limited physical exercise [9,10,11]) and to conditions such as diabetes mellitus, hypertension, hyperlipidemia, metabolic syndrome, depression, and lower urinary tract symptoms [12,13].

In COPD patients, the presence of endothelial dysfunction, smoking, chronic hypoxia, cardiovascular comorbidities, hormonal imbalance, and mood disorders related to status and medications can limit the pursuance of penile erection and subsequently lead to an unsatisfactory sexual life [14,15]. This issue can impair overall QoL, a parameter often compromised in COPD men [16,17].

In a pneumological setting, little time of the examination is dedicated to sexual issues, even though the latest guidelines consider assessment of sexual activity as a necessary part of the evaluation in COPD men [1]. Unfortunately, these guidelines do not include ED as one of the possible comorbidities of COPD [18]. A previous study demonstrated that 87% of patients with COPD do not discuss their sexual problems with their physicians and that 78% do not share them with their wives [19]. Moreover, sexuality is still a taboo subject, especially in some Asian cultures, where sexual issues are an improper topic. For example, in Korea, only 2% of men discuss their intimate issues [20].

At the present, few studies have investigated ED in patients with COPD, and this topic remains a neglected area of research.

The aim of this narrative review is to investigate sexual function and ED in COPD men.

## 2. Sexual Function in COPD Patients

Sexuality is a lifelong necessity in order to pursue human well-being [21,22]. Sexual function is a health status and QoL indicator. Factors including ageing, physical and psychological problems, associated morbidities, medications, and generally poor health can cause diminished sexual activity, low libido, and ED [23].

Living with a chronic disease, such as COPD, disrupts the natural course of daily life and can cause deterioration in sexual life [22,24]. Respiratory or general symptoms of COPD contribute to sexual dysfunction via somatophysical effects: dyspnea, cough, muscular weakness, and the associated reduction of physical activity are among the major causes of decreased sexual activity in these patients [22,25].

Sexual performance causes an increase in tidal volume and breathing frequency, associated with an increased cardiopulmonary load [26]. Energy spent during sexual intercourse is equal to the energy required for walking with a speed of 5–6 km/h or continuous stair climbing for 3–4 minutes [14,27]. Sexual activity may exceed the respiratory capabilities of patients with severe airway obstruction. Ventilatory demands may force a passive approach or avoidance, supporting isolation, denial, or repression [25,28].

QoL generally diminishes in COPD as the disease progresses [29]. Moreover, sexual dysfunction negatively affects QoL [14]. ED is usually the most common sexual dysfunction experienced [14] and is one of the main reasons for a decrease in QoL of men and their partners.

ED has a profound negative impact on men’s QoL and self-esteem. Several studies have reported ED as a common comorbidity in patients with COPD. However, sexual history is usually neglected or avoided as health professionals are poorly trained in collecting this kind of information, compared with their expertise in obtaining information about physiological functions [28,30].

The latest GOLD guidelines [1] ask physicians to assess the state of sexual activity in COPD men, but they do not contain any information regarding how to evaluate or manage sexual dysfunction. Consequently, clinicians who follow these guidelines usually do not ask patients about sexual complaints. This is an important limitation to patients’ overall QoL assessment. It was reported that in COPD patients, physicians usually focus on bronchospasm, respiratory failure, and infections, while sexual problems receive less attention [24,31].

Other studies showed that COPD patients do not commonly share their sexual issues with their physicians [19,23,24].

## 3. ED and COPD Men

A satisfactory erection is one of the main aspects of a healthy and satisfactory male sexual life. In Europe, about 30% of men aged 40 to 79 years report suffering from ED, and the percentage increases with age [32].

In COPD men, studies reported a higher prevalence of ED [14,18,33,34,35], ranging from 72% to 87% of cases, with the severe disease stage related to poorer erectile function [34]. Furthermore, a recent meta-analysis performed by Luo et al. [36], involving about 58,000 patients, highlighted a higher prevalence of moderate and severe ED among patients with COPD. Nevertheless, the exact pathogenesis of ED in COPD men remains poorly understood. The contribution of four main elements is required to achieve a proper erection: adequate testosterone levels (hormonal factor), a sufficient blood supply (vascular factor), proper sexual stimuli (psychological factor), and suitable innervation (neurological factor) [37]. Although the nervous system is usually not compromised in COPD patients, the other three elements can be causative of ED. The definitive mechanism of a more widespread and severe ED development in COPD men remains unknown, but it is thought to be multifactorial (Appendix A Table A1).

## 4. Pathogenesis of ED in COPD

### 4.1. Hormonal Factor

Androgen deficiency can lead to decreased libido and difficulty with penile erections [38]. Lower testosterone levels have been reported in men with COPD [4,36,39,40,41,42]. In particular, Karadag et al. [4] found lower testosterone levels in 95 COPD men (compared with controls) and a positive correlation between partial pressure of oxygen in arterial blood and serum testosterone. Furthermore, they demonstrated a significant rise in serum LH and FSH and an important decline in testosterone levels during COPD exacerbation.

Nonetheless, in a study by Kahraman et al. [40], the decline in testosterone levels was insignificant in COPD patients compared with the controls, but serum LH, FSH, and estradiol levels were significantly higher in COPD men.

A link between gonadal status and sexual dysfunction in men with COPD is probable. As COPD is an illness sustained by chronic and systemic inflammation arising from the lungs, it may possibly extend to the testes, leading to decreased testosterone production.

When inflammation occurs, inflammatory cytokines are synthesized by different cells, such as immune cells, endothelial cells, arterial smooth muscle cells, and adipocytes as a multi-system response to any harmful stimulus [43]. Cytokines are known to provide significant regulatory functions for steroidogenesis in Leydig cells. However, acute and chronic inflammation are associated with inhibition of testosterone synthesis and transient or chronic male hypogonadism. Changes in serum cytokines may have direct detrimental effects on the hypothalamus–pituitary–gonadal axis, negatively affecting testes function, as reported in chronic inflammatory disease [44,45,46].

The presence of systemic inflammation and its involvement in testosterone production may explain the high prevalence and increased severity of ED in COPD. The relationship between ED and serum TNF-α concentration, which is a known marker of systemic inflammation in COPD, was investigated [22]. Serum TNF-α concentration was significantly higher in COPD patients with moderate-to-severe ED, suggesting that chronic inflammation is likely to play a role in ED in these patients. Whether TNF-α has a direct effect on ED or is a reflection of more severe COPD is still not clear, owing to a lack of additional studies in COPD men on the relationship between ED and pro-inflammatory cytokines [22].

### 4.2. Vascular Factor

COPD and cardiac comorbidities are frequently associated and share common risk factors, pathophysiological processes, clinical signs, and symptoms [47]. Pulmonary hypertension (PAH), right ventricular dysfunction, dysrhythmia, and ischemic coronary disease are known severe consequences of COPD progression [48,49]. Patients with COPD are particularly vulnerable to cardiac disease, with a higher incidence and prevalence (age- and sex-adjusted) compared with patients without COPD [50]. Not surprisingly, COPD has thus been associated with ED development.

It must be considered that cardiovascular risk factors, in an environment of systemic inflammation, are linked to endothelial dysfunction and atherosclerosis [51]. These pathogenetic mechanisms are shared between cardiovascular diseases and ED [52]. Endothelial dysfunction plays a major role in the pathogenesis of ED and coronary artery disease [53,54]. Low-grade subclinical inflammation affects endothelial function and might lead to a prothrombotic status. Several studies reported that ED onset and severity are associated with increased expression of inflammation markers and endothelial/prothrombotic factors [54,55,56,57]. This setting may predispose the patient to the rupture of unstable coronary plaques and occurrences of acute coronary events [51].

Atherosclerosis involves the whole circulatory system, but the first compromised vessels are the smaller ones, such as the penile (1–2 mm) and the coronary (3–4 mm) arteries (artery-size hypothesis) [58,59]. Larger vessels better tolerate the same amount of endothelial dysfunction and/or atherosclerotic burden compared with smaller ones. The corresponding quantity of damage may lead to a more significant blood flow reduction in erectile tissues than in coronary arteries [58]. Thus, the penile vascular bed can be a sensitive indicator of systemic vascular diseases [51]. According to epidemiologic and clinical evidence, ED has been reported to predate the occurrence of cardiovascular disease symptoms by about 2–5 years [60,61]. There is a wealth of evidence demonstrating that ED is an independent risk factor of cardiovascular diseases [62,63,64,65]. Dong et al. performed a meta-analysis of 12 prospective studies and reported evidence of an independent association of ED with increased risk of major cardiovascular events and all-cause mortality [9]. Uddin et al. [62] assessed the utility of self-reported ED for predicting cardiovascular events in 1914 male participants (mean age, 69 ± 9.2 years) of the Multi-Ethnic Study of Atherosclerosis, followed over almost 4 years. They found self-reported ED to be a significant predictor of major cardiovascular events (hazard ratio 1.9) after adjustment for established cardiovascular risk factors, depression, and use of beta-blockers.

Smoking is another risk factor for ED in COPD men. It is well-known that smoking is the leading cause of COPD; furthermore, smoking can induce ED through multiple pathways: reduction in releasing nitric oxide (NO) by loss of neuronal-nitric oxide synthase (n-NOS) and endothelial-NOS [66], direct damage to tissues [67,68], and direct production of superoxide anions [69]. Furthermore, smoking causes intrinsic damage to vessels, preventing elastic dilation [70]. Turan et al. [17] demonstrated a negative correlation between worse erectile performance, assessed by a specific score (International Index of Erectile Function Questionnaire–IIEF score), and the amount of smoking in COPD patients.

Smoking and COPD disease were found to be independent risk factors for ED [71].

In addition, chronic hypoxia is an important risk factor associated with pathological conditions, including ED. A couple of trials have shown that inadequate oxygen supply impairs NO synthesis, which subsequently reduces the functional integrity of penile smooth muscles [72,73]. It is suggested that ED in COPD may be due to persistent exposure to a hypoxic environment [74] in which endothelial dysfunction and vascular insufficiency may also play an important role. Chronic hypoxia affects the pulmonary vasculature through both tonic vasoconstriction and vascular remodeling with myointimal hyperplasia of the pulmonary vascular bed [75]. Hypoxia from decreased corpora cavernosa oxygenation can cause a decrease in prostaglandin E1 levels, which normally inhibit pro-fibrotic cytokines, such as transforming growth factor β1. These profibrotic cytokines promote collagen deposition, replacing the smooth muscle and resulting in decreased elasticity of the penis, as was shown in several rat models. As the smooth muscle to collagen ratio decreases and collagen content increases, the ability of the corpora cavernosa to compress the subtunical veins decreases, leading to corporal veno-occlusive dysfunction [8].

### 4.3. Psychological Factor

A recent meta-analysis conducted by Liu et al. [76] stated that the risk of ED increases by 39% in patients with depression and that the incidence of ED is 1.39 times higher in depressed patients than in those who are not. In COPD patients, higher depression scores were related to poor or absent sexual function [77].

Anxiety and depression are among the most common comorbidities in COPD [78]. A large study by Hanania et al. [79] highlighted a prevalence of depression in 2120 COPD patients of about 26% compared with 12% in the controls. These results are similar to those of Janssen et al. [80] and Turan et al. [81].

The prevalence of clinical anxiety in COPD outpatients ranges between 13% and 46% [82]. Eisner et al. [83] reported that COPD patients are 85% more likely to develop anxiety disorders compared with healthy, matched controls.

The exact mechanisms linking COPD with depression and anxiety have not been fully identified [24] and the associations are likely to be bidirectional [84]. Androgen deficiency may be an additional causal factor: it can induce depression, anxiety, anger, fatigue, and sleep disorders [85]. Furthermore, depression and anxiety may lead to fear, panic and hopelessness, low self-esteem, social isolation, and dependence on caregivers, initiating a vicious cycle that perpetuates anxiety and depression [86].

Emerging evidence suggests that low-grade chronic inflammation partially mediates the association of depressive symptoms and pulmonary function. Increased inflammatory markers have been documented in both late-life depression [87] and COPD [88].

It was demonstrated that in patients with COPD, left ventricular dysfunction has a negative impact on exercise tolerance, being associated with anxiety and depression, reduced carbon monoxide diffusion, and higher prevalence of right ventricular dysfunction. Similarly, physical activity impairment imposed by possible comorbidities of COPD patients can impair sexuality and can consequently worsen QoL [89].

To conclude, Kahraman et al. [40] found a strong significant correlation between hypoxemia and depression and between hypoxemia and ED in COPD patients.

### 4.4. Medications: Possible Influence on ED

Several drugs are associated with an increased risk of ED developing. Until now, there are no proper studies involving COPD men linking medication and ED incidence.

However, considering male COPD status and possible comorbidities, we highlight:

- Specific drugs used for COPD as anticholinergics, usually called SAMA (short-acting muscarinic antagonists) and LAMA (long-acting muscarinic antagonists) were associated with ED, up to an odd ratio of 12.8, in a study by Ricci et al. [90]. Corticosteroid treatment is commonly used for short-term periods during COPD exacerbations [1] and may have an indirect effect on ED incidence: glucocorticoids alter hypothalamus–pituitary–gonadal axis function, inhibiting GnRH synthesis and consequently LH release [91,92]. This leads to a relative hypogonadal status. Major evidence is linked to the use of glucocorticoids in supraphysiological doses: prednisone in daily doses of 15 mg or higher has been reported to suppress serum testosterone concentrations in a dose-dependent manner [93,94]. Moreover, the use of high doses of corticosteroids for prolonged periods of time may increase atherosclerotic plaque burden, as observed in patients with Cushing’s syndrome [95]. This can hypothetically represent an additional pathogenetic factor for vascular ED.
- Considering the high prevalence of mood disorders, antipsychotic and antidepressant drugs (in particular typical antipsychotics, tricyclic antidepressants, selective serotonin-reuptake inhibitors, and benzodiazepines) can induce ED and loss of libido [96,97].
- Considering the high frequency of cardiovascular comorbidities, the use of antihypertensive drugs such as beta-blockers, thiazide diuretics, and spironolactone can induce ED [90,97].

## 5. Management of ED in COPD Men

No data are currently available regarding proper ED therapy in COPD men. The management of ED in these patients can be adapted from the general published guidelines [98]. Initial treatment is usually based on lifestyle modifications (as much as possible, considering airflow limitation and cardiac comorbidities) and correction of predisposing factors (hypogonadal status, mood disorders, and medications) [8]. Accordingly, the European Association of Urology states that “lifestyle changes and risk factor modification must precede or accompany any ED treatment” and classifies the level of evidence as 1b with a grade A recommendation [98]. Targeting smoking, alcohol consumption, obesity, and limited physical activity can significantly improve the quality of erections by reducing endothelial dysfunction, insulin resistance, and an inflammatory state [99,100,101,102,103,104,105,106,107,108]. In particular, Gupta et al. performed a meta-analysis that assessed the effects of lifestyle modifications and the reduction in cardiovascular risk factors on the severity of ED. Their findings showed incremental benefits on erectile function regardless of other therapies on ED [108].

Testosterone replacement therapy is recommended in men with ED who have low concentrations of bioavailable testosterone. In a meta-analysis by Rademaker et al., improvement in ED was significantly more common in men with hypogonadism who were treated with testosterone than in those who received placebo (57% vs. 17%) [109].

Psychological and psychosexual therapies can be indicated if the patient suffers from significant psychological distress. While psychological support has strong evidence of improving mood status [110,111], data regarding the efficacy of psychosexual techniques (sensate focus, sex education, and interpersonal therapy) are largely inconclusive [37].

Eventually, a review of the patient’s medications might reveal drugs that interfere with a proper erection.

First-line pharmacological therapies in ED include oral phosphodiesterase 5 inhibitors (PDE5-i) and vacuum erection devices. Second-line therapies consist of an intraurethral suppository of prostaglandin E1 (alprostadil) and intracavernosal injection with vasoactive substances. A penile prosthesis is usually reserved as a final option [8].

Oral PDE5-i are beneficial in correcting ED in a wide range of patients with varying etiologies of sexual dysfunction and they seem to be a reasonable first-line drug therapy in COPD men. They include sildenafil, vardenafil, tadalafil, and avanafil (all available in Europe and the United States). These drugs competitively inhibit the PDE5 enzyme, leading to relaxation of the corpus cavernosum smooth muscle cells and subsequently promoting an erection [112]. PDE5-i still require sexual stimulation, both physical and mental, to create arousal [113]. They have generally mild side effects (headache, flushing, and myalgia). However, caution must be taken in patients with coronary artery disease, heart failure, or taking certain drugs that can worsen side effects (nitrates, α adrenergic receptor blockers).

Another indication to use PDE5-i (sildenafil and tadalafil) is PAH, a common cardiovascular comorbidity associated with COPD [114]. It is defined as a condition of increased pulmonary arterial resistance, which leads to an elevated pulmonary arterial pressure (>25 mmHg at rest) that endangers the heart and causes right-sided heart failure [115]. It occurs mainly due to endothelial dysfunction and vascular smooth muscle hypertrophy [116,117]. Sildenafil in PAH has shown the capability to suppress inflammation and prevent the remodeling of pulmonary vascular bed [118].

Moreover, it was demonstrated that sildenafil reduces inflammatory blood mediators, such as interleukin-6, C-reactive protein, fibrinogen, and tumor necrosis factor-α, including in patients with vasculogenic ED [119].

Finally, a recent meta-analysis by Hao et al. showed that the use of sildenafil is effective and safe in COPD men with PAH [120]. Further studies are needed to prove additional beneficial effects of PDE5-i on ED in COPD men with PAH.

## 6. Conclusions and Perspectives

Sexual dysfunction is a common finding in COPD men. Hypoxemia, smoking, lower testosterone levels, cardiovascular diseases, limitation of physical activity, and psychological distress are thought to be the foremost mechanisms associated with ED in these patients. The presence of ED negatively affects the functional capacity and QoL in COPD men.

Sexual dysfunctions cannot be treated until a proper history defines the problem. Open communication between the physician and the patient is essential for successful management of the issue. However, sexual history is usually neglected or avoided as healthcare professionals are poorly trained to obtain it. Greater knowledge of this topic among physicians may help COPD patients to cope with the impact of the disease not only in their daily lives but also in their sexuality. This may improve overall QoL.

Furthermore, additional studies are needed to assess the most appropriate therapeutic strategies to improve sexual function in these men.

## Data Availability

Not applicable.

## Appendix A

**Table A1:** Presumed Causes of ED in COPD Men.

Factor	Evidence
Hormonal imbalance	lower testosterone levels due to chronic inflammation (impairment of Leydig cells and HPG axis) [44,45,46]
Vascular alterations	endothelial dysfunction and atherosclerosis (impaired vascularization) [52,53,54,55,56,57] smoking habit (reduced nitric oxide production, direct damage to vessels) [66,67,68,69,70] chronic hypoxia (vascular remodeling) [7,74,75]
Psychological status	depressive mood and anxiety (inflammation, androgen deficiency, impairment in physical activities, hypoxemia) [40,85,86,87,88,89]
Medications	anticholinergics (SAMA, LAMA) [90] glucocorticoids [91,92,93,94,95] psychotropic drugs (typical antipsychotics, tricyclic antidepressants, selective serotonin-reuptake inhibitors, and benzodiazepines) [96,97] anti-hypertensive drugs (beta-blockers, thiazide diuretics, and spironolactone) [90,97]

ED: erectile dysfunction; COPD: chronic obstructive pulmonary disease, HPG: hypothalamus pituitary gonadal, SAMA: short-acting muscarinic antagonists, LAMA: long-acting muscarinic antagonists.
